# *Filifactor alocis *- involvement in periodontal biofilms

**DOI:** 10.1186/1471-2180-10-66

**Published:** 2010-03-01

**Authors:** Sebastian Schlafer, Birgit Riep, Ann L Griffen, Annett Petrich, Julia Hübner, Moritz Berning, Anton Friedmann, Ulf B Göbel, Annette Moter

**Affiliations:** 1 Institut für Mikrobiologie und Hygiene, Charité - Universitätsmedizin Berlin, Dorotheenstraße 96, 10117 Berlin, Germany; 2 Abteilung Parodontologie, Centrum für Zahn-, Mund- und Kieferheilkunde, Charité - Universitätsmedizin Berlin, Aßmannhauser Straße 4-6, 14197 Berlin, Germany; 3 Section of Pediatric Dentistry, College of Dentistry, Ohio State University, 305 W. 12th Avenue, Columbus, Ohio 43210, USA

## Abstract

**Background:**

Bacteria in periodontal pockets develop complex sessile communities that attach to the tooth surface. These highly dynamic microfloral environments challenge both clinicians and researchers alike. The exploration of structural organisation and bacterial interactions within these biofilms is critically important for a thorough understanding of periodontal disease. In recent years, *Filifactor alocis*, a fastidious, Gram-positive, obligately anaerobic rod was repeatedly identified in periodontal lesions using DNA-based methods. It has been suggested to be a marker for periodontal deterioration. The present study investigated the epidemiology of *F. alocis *in periodontal pockets and analysed the spatial arrangement and architectural role of the organism in *in vivo *grown subgingival biofilms.

**Results:**

A species-specific oligonucleotide probe, FIAL, was designed and evaluated. A total of 490 subgingival plaque samples were submitted to PCR and subsequent dot blot hybridization to compare the prevalence of *F. alocis *in patients suffering from generalized aggressive periodontitis (GAP), chronic periodontitis (CP), and control subjects resistant to periodontitis. Moreover, a specially designed carrier system was used to collect *in vivo *grown subgingival biofilms from GAP patients. Subsequent topographic analysis was performed using fluorescence in situ hybridization.

While the majority of patients suffering from GAP or CP harboured *F. alocis*, it was rarely detected in the control group. In the examined carrier-borne biofilms the organism predominantly colonized apical parts of the pocket in close proximity to the soft tissues and was involved in numerous structures that constitute characteristic architectural features of subgingival periodontal biofilms.

**Conclusions:**

*F. alocis *is likely to make a relevant contribution to the pathogenetic structure of biofilms accounting for periodontal inflammation and can be considered an excellent marker organism for periodontal disease.

## Background

Periodontitis is a chronic inflammatory bacterial infection leading to destruction of periodontal ligaments and supporting bone of the tooth. Its aetiology has been a field of intensive research in the past decades. As periodontal pockets accommodate a multitude of bacterial phylotypes, it is difficult to differentiate between mere commensals and true pathogens. During the 1970's, 80's and early 90's, research focused mainly on a number of culturable bacteria like *Porphyromonas gingivalis, Prevotella intermedia, Aggregatibacter *(*Actinobacillus*)*actinomycetemcomitans, Tannerella forsythia *and *Treponema denticola *that proved to be associated with the disease [1]. Studies have determined their relative prevalences, interactions and virulence factors [2-7]. By the end of the 1980's, the development of novel, culture-independent techniques allowed the identification of as-yet-unculturable and fastidious organisms in patients suffering from periodontitis and added new insight into bacterial communities in periodontal pockets [8-10]. In recent years, research has detected increasing numbers of bacterial species and phylotypes in subgingival plaque and other habitats of the human oral cavity [11-18]. There is little reason to believe that easily culturable bacteria contribute more to the development of periodontitis than fastidious organisms. Doubt has been raised whether the widely accepted periodontal pathogens *P. gingivalis, P. intermedia *and *T. forsythia *are appropriate diagnostic markers to differentiate between health and disease [19,20].

Along with these discoveries it became clear that the mere isolation and characterization of bacteria from diseased sites is not a sufficient approach to understand the complex pathogenesis of periodontitis. The organisms do not live in a planktonic form, but rather as a sessile community attached to the tooth surface in a matrix of extracellular polymers [21]. The structure and function of these bacterial biofilms are influenced both by bacterial interactions and host factors. Exploring the biofilm architecture and identifying its bacterial architects are pressing goals in current periodontal research.

*Filifactor alocis *(ATCC 35896^T^) was first isolated in 1985 from the human gingival crevice as *Fusobacterium alocis *[22] and later reclassified as *Filifactor alocis *[23]. It is a fastidious, Gram-positive, obligately anaerobic rod that possesses trypsin-like enzymatic activity [24], as do *P. gingivalis *and *T. denticola *[25,26]. In recent years, it has been discovered in patients suffering from chronic periodontitis (CP) [14,18,27,28], generalized aggressive periodontitis (GAP) [29] and endodontic infections [30]. Recently, *F. alocis *was detected in elevated numbers in CP patients with periodontal deterioration compared to patients with a stable periodontal condition and was therefore proposed as a potential marker for active disease [19].

The present study chose a DNA-based epidemiological approach utilizing dot blot hybridization to investigate the prevalence of *F. alocis *in subjects with GAP, CP, and in a subject group resistant to periodontitis. Furthermore, fluorescence in situ hybridization (FISH) was employed to analyse the spatial arrangement and the architectural role of *F. alocis *in periodontal pockets. For that purpose, a specially designed carrier system was used to collect *in vivo *grown biofilms from GAP patients [31].

## Methods

### Oligonucleotide probes

To detect *F. alocis*, a species-specific probe, FIAL (5'-TCTTTGTCCACTATCGTTTTGA-3') was designed after comparative sequence analysis of close phylogenetic neighbours to *F. alocis*. To ensure specificity, the probe sequence was compared to the sequences deposited in the Ribosomal Database Project II [32] and to all 16S rRNA entries at the EMBL and GenBank databases (as of August 2009) employing the Husar program package (DKFZ, Heidelberg, Germany). The probe was checked for its practical use in hybridization experiments with the program OLIGO (version 4.0). EUB 338, a probe complementary to a highly conserved region of the 16S rRNA gene in bacteria, was used in dot blot hybridization experiments to verify successful PCR amplification and in FISH experiments to detect and visualize large parts of the bacterial biofilm population [33]. For comparative purposes, probes POGI, PRIN, ACAC, TDEN, FUNU and B(T)AFO were employed in dot blot experiments to detect *P. gingivalis*, *P. intermedia*, *A. actinomycetemcomitans*, *T. denticola*, *Fusobacterium nucleatum *and *T. forsythia*, respectively. These probes have been published previously and deposited in ProbeBase [34].

### Clinical samples for dot blot hybridization

A total of 490 subgingival plaque samples from 121 patients were examined and evaluated. Samples from GAP and CP patients were obtained from those reporting to the departments of periodontology of the Charité - Universitätsmedizin Berlin, the Dresden University of Technology, the University of Oslo and the University of Basel. These patients were diagnosed according to the criteria of the 1999 International Workshop for the Classification of Periodontal Diseases and Conditions [35] (see Table 1). Control samples were taken from elderly patients of a private periodontal practice in Berlin. These subjects, aged 65 years and older, had at least 20 natural teeth and displayed only mild periodontal disease. They had not received periodontal treatment previously, exhibited no sites with attachment loss of more than 2 mm or probing pocket depth (PPD) of more than 5 mm and will be referred to as periodontitis resistant (PR) patients in the following. Subjects suffering from chronic systemic disease were excluded from the study as well as pregnant or breast feeding women and patients who had received antiinflammatory or antimicrobial therapy within the past six months. Patient demographics are presented in Table 2. Ethical approval was given by the Ethical Committee at Charité - Universitätsmedizin Berlin. All patients signed informed consent forms. After removal of supragingival plaque the deepest periodontal pockets available were sampled. In GAP patients, additional samples were taken from shallow sites if present. None of the samples were taken from the same site in one patient. Three sterile paper points (ISO 35, Becht, Offenburg, Germany) were inserted into the pockets, removed after 10 seconds and placed immediately in 1 ml of reduced transport fluid (RTF) [36] containing 25% glucose.

**Table 1:** Clinical criteria for patient selection

Periodontitis Resistant (PR) subjects	Age ≥ 65 years
	≥ 20 natural teeth
	Probing Depth at any site ≤ 5 mm
	Clinical Attachment Loss at any site ≤ 2 mm
Chronic Periodontitis (CP)	≥ 4 mm Probing Depth at ≥ 30% of residual teeth

Generalized Aggressive Periodontitis (GAP)	Disease onset estimated at < 30 years based on clinical examination, past radiographs, and/or interview
	≥ 6 mm Probing Pocket Depth at > 3 permanent teeth other than first molars and incisors

**Table 2:** Patient demographics

Clinical samples processed by dot blot hybridization						
**Subject ** **group**	**No. of ** **patients**	**Age (yr) ** **± SD**	**Gender**		**Plaque samples**	

			**f**	**m**	**n**	**mean PPD ** **(mm) ± SD**

GAP	72	34.8 ± 6.4	45	27	330	7.8 ± 2.5

CP	30	51.0 ± 10.2	15	15	78	7.1 ± 1.4

PR	19	66.7 ± 1.5	12	7	82	3.6 ± 0.8

**Clinical samples for FISH**						

**Subject ** **group**	**No. of ** **patients**	**Age (yr) ** **± SD**	**Gender**		**Carrier samples**	

			**f**	**m**	**n**	**mean PPD ** **(mm) ± SD**

GAP	11	34.3 ± 7.9	5	6	28	8.1 ± 1.7

### Dot blot hybridization

**Figure 1:**
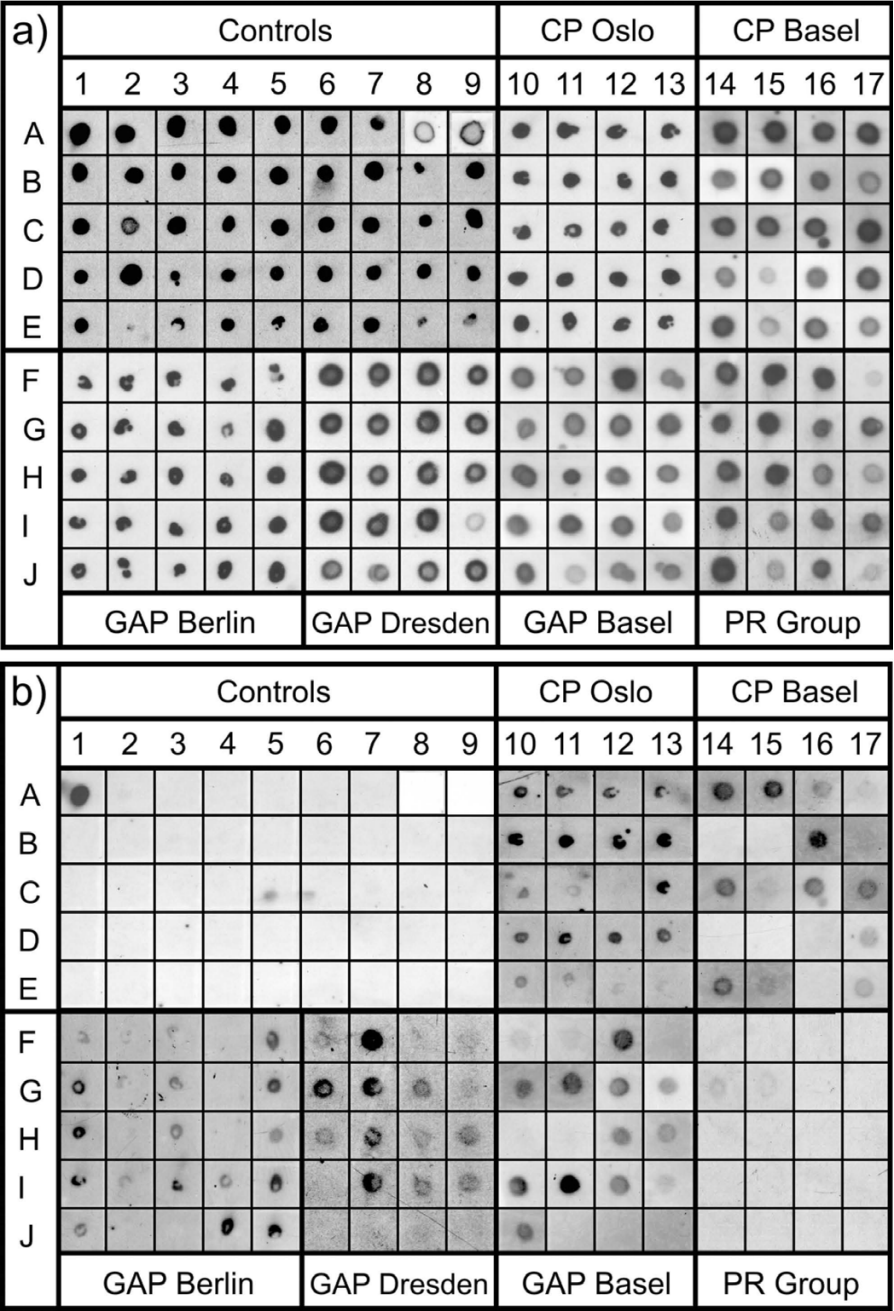
**Dot blot hybridizations of identical membranes with EUB 338 (a) and the species-specific probe FIAL (b)**. PCR-amplified products from *F. alocis *(field A1) and its closest cultured relative *F. villosus *(A2) served as positive and negative controls, respectively. Additionally, products from the following bacteria were applied as negative controls: *Centipeda periodontii *(DSM 2778) (A3), *Selenomonas noxia *(DSM 19578) (A4), *Selenomonas ruminantium *(DSM 2150) (A5), *Selenomonas lacticifex *(DSM 20757) (A6), *Selenomonas sputigena *(DSM 20758) (A7), *Eggerthella lenta *(ATCC 25559) (A8), *Peptostreptococcus anaerobius *(ATCC 27337) (A9), and *Actinomyces viscosus *(ATCC 15987) (B1), *Streptococcus intermedius *(ATCC 27335) (B2), *Streptococcus mutans *(ATCC 35668) (B3), *Neisseria lactamica *(ATCC 23970) (B4), *Flavobacterium odoratum *(ATCC 4651) (B5), *Fusobacterium necrophorum *(NCTC 25286) (B6), *Fusobacterium periodonticum *(CCUG 14345) (B7), *Fusobacterium simiae *(CCUG 16798) (B8), *F. nucleatum *(ATCC 25586) (B9), *Klebsiella pneumoniae *(ATCC 23357) (C1), *Veillonella dispar *(ATCC 17748) (C2), *Veillonella parvula *(ATCC 10790) (C3), *Kingella kingae *(ATCC 23330) (C4), *Eikenella corrodens *(CCUG 2138) (C5), *Bacteroides fragilis *(ATCC 25285) (C6), *Bacteroides gracilis *(ATCC 33236) (C7), *Campylobacter concisus *(ATCC 33236) (C8), *Campylobacter rectus *(ATCC 33238) (C9), *Capnocytophaga gingivalis *(ATCC 33624) (D1), *Capnocytophaga sputigena *(ATCC 33612) (D2), *Capnocytophaga ochracea *(ATCC 27872) (D3), *Prevotella buccalis *(ATCC 33690) (D4), *Prevotella oralis *(MCCM 00684) (D5), *Prevotella nigrescens *(NCTC 9336) (D6), *Porphyromonas asaccharolytica *(ATCC 25260) (D7), *P. intermedia *(ATCC 25611) (D8), *P. gingivalis *(ATCC 33277) (D9), *Haemophilus paraphrophilus *(ATCC 29241) (E1), *Haemophilus aphrophilus *(NCTC 55906) (E2), *Haemophilus influenzae *(clinical isolate) (E3), *Haemophilus influenzae *(ATCC 33391) (E4), *Pasteurella haemolytica *(ATCC 33396) (E5), *Leptotrichia buccalis *(MCCM 00448) (E6), *A. actinomycetemcomitans *(MCCM 02638) (E7), *A. actinomycetemcomitans *(ATCC 33384) (E8) and *A. actinomycetemcomitans *(ATCC 43718) (E9). In columns 10-17 and in lanes F to J of columns 1-9 PCR products from patient samples of the different diseased groups and the periodontitis resistant (PR) group were applied. (a): Signals in all fields prove successful PCR-amplification. (b): Absence of signals in all bacterial controls along with strong signal in field A1 proves specificity of the experiments. Prevalences of *F. alocis *in all diseased collectives exceed the prevalence in the PR group.

DNA extraction from the 490 collected subgingival plaque samples, subsequent PCR amplification, preparation of dot blot membranes and dot blot hybridization experiments to analyse the prevalence of *F. alocis *were performed as published previously [37]. The broad range bacterial primers TPU1 5'-AGAGTTTGATCMTGGCTCAG-3' (corresponding to complementary positions 8-27 in the *Escherichia coli *16S rRNA gene) and RTU3 5'-GWATTACCGCGGCKGCTG-3' (corresponding to positions 519-536 in *E. coli *16S rRNA) were used to amplify part of the 16S rRNA gene out of the bulk DNA. Agarose gel electrophoresis confirmed successful amplification. Hybridizations with both EUB 338 and FIAL were carried out at 54°C, while stringency washes were performed at 58°C for EUB 338 and at 60°C for FIAL with a washing buffer containing 2× SSC (1× SSC is 0.15 M NaCl plus 0.015 M sodium citrate) - 0.1% SDS for EUB 338 and 5× SSC - 0.2% SDS for FIAL. In all experiments, PCR-amplified products obtained from fixed cells of *F. alocis*, its closest cultured phylogenetic relative *Filifactor villosus *(ATCC 33388^T^), and a panel of 43 periodontal pathogens (see Figure 1 legend) and related bacteria were included as positive and negative controls, respectively. After hybridization, X-ray films were exposed for 2 to 30 hours. After stripping, all membranes were re-used for further experiments.

### Statistical analysis

Statistical evaluation of the dot blot hybridization results was performed using the exact chi-square test. The prevalence of *F. alocis *in different patient groups was compared. Moreover, the presence of *F. alocis *in relation to the PPD was analysed. P values below 0.05 were considered statistically significant.

### Clinical samples for FISH

A carrier system designed to collect biofilms grown *in vivo *in periodontal pockets was used for sampling [31]. Ethics approval for subgingival sample collection was given by the Ethical Committee at Charité - Universitätsmedizin Berlin. Expanded polytetrafluoroethylene (ePTFE) membranes were placed in periodontal pockets of GAP patients for 7 to 14 days and colonized by the subgingival bacterial flora. Strips of ePTFE measuring 3 mm in width were wrapped around and attached to rigid plastic tips (Plast-O-Probe; Maillefer, Ballaigues, Switzerland), which permitted the insertion of these strips down to the bottom of the pocket, therefore allowing the complete extension of the membrane over the entire probing depth. One side of the double bent strip faced the soft tissue and the other side, slightly longer, faced the root surface. This longer cervical end was fixed to the tooth with cyanoacrylic glue (Tesa, Beiersdorf, Hamburg, Germany) to stabilize the position of the carrier. After removal, carriers were fixed for at least 3 h with 3.7% (v/v) formaldehyde in phosphate-buffered saline (pH 7.4) and embedded in cold polymerizing resin (Technovit 8100, Kulzer, Wehrheim, Germany) as reported previously [38]. Sectioning into slices of 2-3 μm was performed as previously published [39]. A total of 28 carriers from 11 GAP patients seeking treatment at the Charité - Universitätsmedizin Berlin were examined. These patients met the same inclusion criteria as the GAP patients selected for dot blot hybridization and likewise signed informed consent forms. See Table 2 for patient demographics. Additionally, a gingival biopsy of a GAP patient obtained during periodontal surgery was processed in the same manner and included in the FISH experiments.

### FISH

FISH experiments were performed as described previously [40] apart from using Vectashield containing DAPI (4,6-Diamidino-2-Phenylindoldihydrochlorid) (Vector Laboratories, Orton Southgate, UK) as mounting medium. The probes were synthesized commercially (biomers.net, Ulm, Germany). EUB 338 was 5' end-labelled with fluorochrome Cy5 (indodicarbocyanine) while FIAL was 5' end-labelled with fluorochrome Cy3 (indocarbocyanine). Differential labelling allowed simultaneous hybridization with both probes.

### Optimization of probe FIAL for FISH

The stringency of FIAL was adjusted by incubating fixed cells of *F. alocis *and its closest cultured relative, *F. villosus *with different hybridization mixes. The formamide concentrations covered a range from 0% (v/v) to 75% (v/v), rising in steps of 5% (v/v). At each level of formamide, a series of images of each bacterial species was taken with a fixed exposure time. The software daime [41] was used to measure the light intensities emitted by both species for each concentration of formamide. While the signal intensity of *F. villosus *did not reach 50 Relative fluorescence Units (RU) at any level of formamide due to unspecific binding of the probe, the intensity of *F. alocis *remained constantly above 150 RU using formamide concentrations of up to 20% (v/v) (see Additional file 1). In addition, fixed cells of 16 different bacterial species, most of them periodontal pathogens, were incubated with FIAL at 20% (v/v) formamide as negative controls, namely *F. nucleatum *(ATCC 25586), *Eikenella corrodens *(CCUG 2138), *Kingella kingae *(ATCC 23330), *Veillonella parvula *(ATCC 10790), *Veillonella dispar *(ATCC 17748), *P. gingivalis *(ATCC 33277), *A. actinomycetemcomitans *(ATCC 33384), *Pasteurella haemolytica *(ATCC 33396), *T. forsythia *(ATCC 43037), *Haemophilus aphrophilus *(NCTC 55906) *P. intermedia *(ATCC 25611), *Campylobacter rectus *(ATCC 33238), *Capnocytophaga sputigena *(ATCC 33612), *Capnocytophaga gingivalis *(ATCC 33624), *Eggerthella lenta *(ATCC 25559), and *Peptostreptococcus anaerobius *(ATCC 27337). As none of the controls were detected by FIAL, all further experiments were performed with 20% (v/v) of formamide, including *F. alocis *as positive and *F. villosus *as negative control.

### Epifluorescence microscopy

After hybridization, carrier and biopsy sections were analysed using an epifluorescence microscope (AxioPlan II, Zeiss, Jena, Germany) equipped with a 100 W high pressure mercury lamp (HBO 103W/2, Osram, Munich, Germany) and 10×, 40× and 100× objectives. DAPI, Cy3 and Cy5 signals were analysed by narrow band filter sets HQ F31-000, HQ F41-007 and HQ F41-008, respectively (AHF Analysentechnik, Tübingen, Germany). Image acquisition was performed with an AxioCam MRm (Zeiss) making use of the AxioVision 4.4 software.

## Results

### Dot blot hybridization

When carried out with the probe EUB 338 (specific for most bacteria), dot blot hybridization experiments indicated the presence of bacteria in all 490 patient samples as well as in the positive (*F. alocis*) and negative controls (see Figure 1 legend) and thus confirmed successful PCR amplification (Figure 1a). The *Filifactor alocis*-specific probe FIAL clearly detected *F. alocis*, while neither the closest phylogenetic neighbour *F. villosus *nor any of the organisms in the panel of oral bacteria (see Figure 1 legend) yielded a signal, thus indicating specific hybridization conditions (Figure 1b).

**Figure 2:**
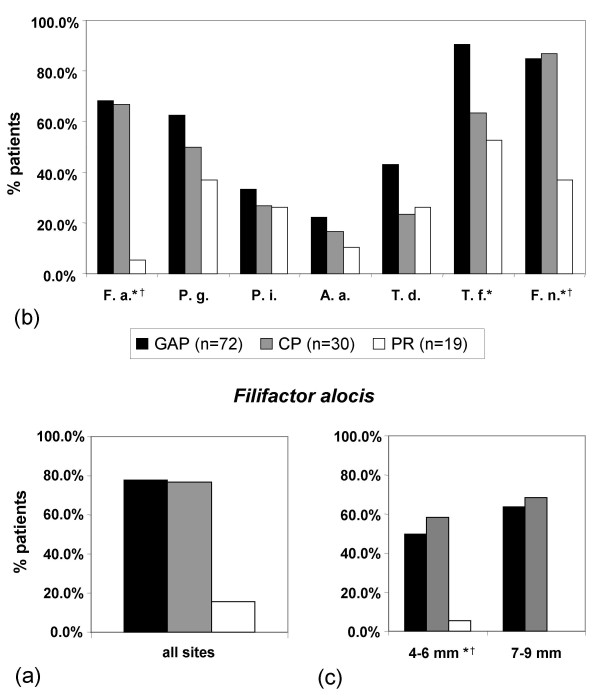
**Prevalence of *F. alocis***. (a): Prevalence of *F. alocis *in all of the samples collected from GAP patients, CP patients and PR subjects as determined by dot blot hybridization using oligonucleotide probes. (b): Prevalence of *F. alocis *(*F. a*.), *P. gingivalis *(*P. g*.), *P. intermedia *(*P. i*.), *A. actinomycetemcomitans *(*A. a*.), *T. denticola *(*T. d.*), *T. forsythia *(*T. f*.), and *F. nucleatum *(*F. n*.) in the deepest pocket of each patient. Asterisks (*) indicate statistically significant differences (p < 0.05) between the GAP and PR groups. Crosses (†) indicate statistically significant differences (p < 0.05) between the CP and PR groups. (c): Percentage of samples positive for *F. alocis *at probing pocket depths 4-6 mm and 7-9 mm. Statistical analysis was limited to one pocket per patient and depth group. Asterisks (*) indicate statistically significant differences (p < 0.05) between the GAP and PR groups. Crosses (†) indicate statistically significant differences (p < 0.05) between the CP and PR groups.

Taking all the collected samples into consideration, *F. alocis *could be identified in 77.8% of the 330 samples from 72 GAP patients, 76.7% of the 78 samples from 30 CP patients and 15.8% of the 82 samples from 19 PR patients (Table 2; Figure 2a). The prevalence of the organism was highest in the Oslo CP collective (87.5%), followed by the Basel GAP collective (80.0%), and the Dresden GAP collective (77.8%) (data not shown). As the number of samples per patient varied between the different collectives, statistical evaluation focused on the deepest pocket of each patient. Prevalence rates were 68.1% for the GAP group, 66.7% for the CP group and 5.3% for the PR group. While detection frequencies did not differ significantly between GAP and CP patients, both diseased groups harboured *F. alocis *significantly more often than the PR group (p < 0.001) (Figure 2b).

The signal intensity of the FIAL-positive patient samples varied between the three groups, suggesting a higher number of *Filifactor *in GAP and CP pockets than in PR pockets tested positive for the organism. Nonetheless, as hybridizations were carried out on PCR-amplified bacterial DNA, no further analysis of signal intensities was performed.

Detection frequencies of *P. gingivalis*, *P. intermedia*, *A. actinomycetemcomitans*, *T. denticola*, *T. forsythia*, and *F. nucleatum *in the three patient groups are displayed in Figure 2b.

To investigate the prevalence of *F. alocis *in relation to the PPD, the donor sites were divided into four groups (I: 1-3 mm, II: 4-6 mm, III: 7-9 mm, IV: > 9 mm). As there is a certain degree of interdependency between pockets belonging to the same patient, statistical analysis was limited to one pocket per patient and probing depth group. Although a slightly higher percentage of group III pockets than group II pockets was positive for *Filifactor *in both the GAP and the CP patients, these differences were not statistically significant. Similarly, analysis revealed no statistically significant differences in the prevalence of the organism in GAP patients compared to CP patients in both pockets of 4-6 mm and pockets of 7-9 mm. In contrast, the prevalence of *F. alocis *in pockets of 4-6 mm differed significantly between both PR and GAP patients (p < 0.001) and PR and CP patients (p < 0.001) (Figure 2c). Insufficient numbers or complete absence of pockets of 1-3 mm in GAP and CP patients, pockets of 7-9 mm in PR patients and pockets deeper than 9 mm in CP and PR patients did not permit further statistical analysis.

### FISH

**Figure 3:**
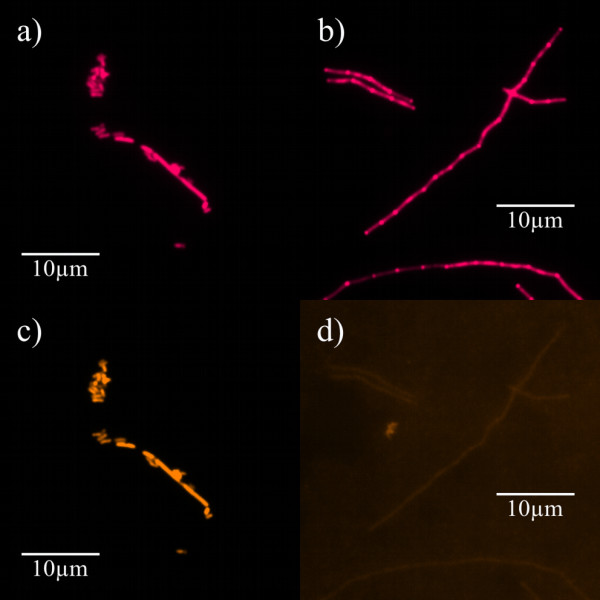
**Specificity of FISH experiments**. Hybridization of fixed cells of *F. alocis *(a and c) and *F. villosus *(b and d) was performed with probes EUB 338-Cy5 (magenta, a and b) and FIAL-Cy3 (bright orange, c and d). (a and c): Identical microscopic fields show detection of *F. alocis *by both EUB 338 (a) and FIAL (c) whereas detection of *F. villosus *by EUB 338 only (b) and not FIAL (d) proves specificity of the FISH experiment.

*F. alocis *was reliably detected by both the species-specific probe FIAL and the eubacterial probe EUB 338. The negative control *F. villosus *was not targeted by FIAL but only by EUB 338, thus confirming specific hybridization conditions (Figure 3). In all of the periodontal ePTFE carriers from GAP patients as well as in the gingival biopsy gained during periodontal surgery, the bacterial biofilms could be visualized by FISH with EUB 338 and displayed characteristic features like densely-packed mushroom-like protuberances and signal-free channels [42]. *F. alocis *could be detected in 9 out of 11 carrier patients (in 17 out of 28 carriers) as well as in the examined gingival biopsy.

**Figure 4:**
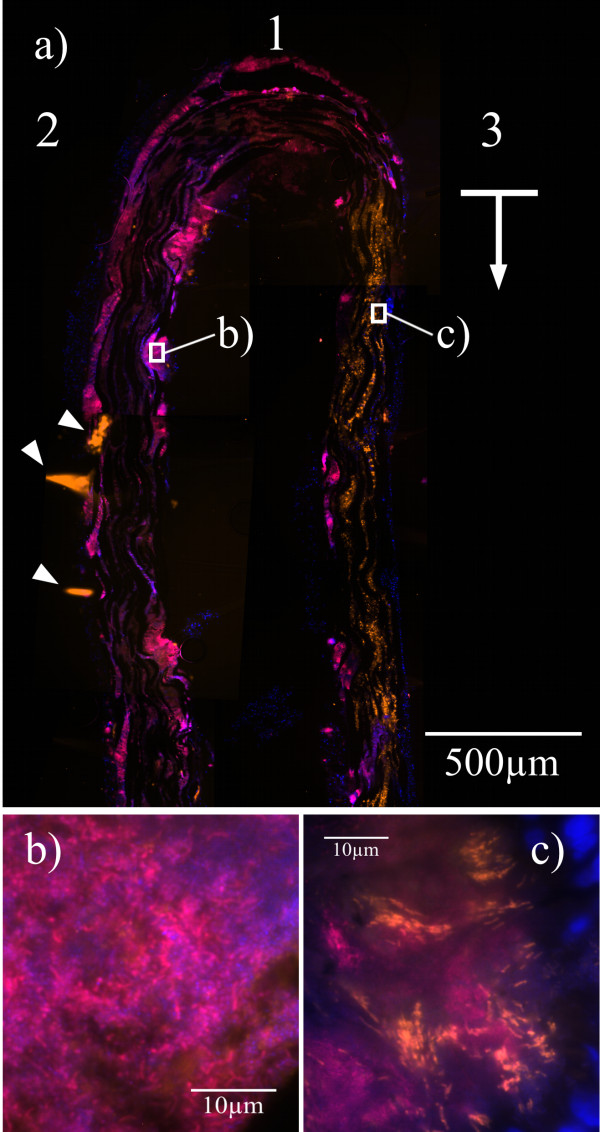
**Carrier grown biofilm visualized by FISH**. Hybridization was performed with the probes EUB 338-Cy5 (magenta) and FIAL-Cy3 (bright orange) along with DAPI staining (blue) on a carrier after 7 days of attachment to the mesial aspect of tooth 16 in a GAP patient. (a): Collage of several microscopic fields in low magnification. The overlay of Cy3, Cy5 and DAPI filter sets shows the bacterial biofilm that grew in the depth of the pocket. EUB 338 visualizes large parts of the bacterial community, while FIAL detects only *F. alocis*. DAPI stains both host cell nuclei and bacteria. The carrier tip (1) and the carrier side facing the tooth (2) show little or no presence of *F. alocis*. The bright orange signal on the carrier side facing the pocket epithelium (3) reveals a strong presence of *Filifactor *in the part of the biofilm indicated by the arrow. Arrowheads on the tooth side (2) point to artifacts caused by upfolding of the embedded carriers. (b and c): Higher magnifications of the inserts. (b) shows the biofilm on the tooth side of the carrier without *F. alocis *among the bacteria. (c) shows *F. alocis *in densely packed groups among the organisms on the epithelium side and host cell nuclei (blue).

**Figure 5:**
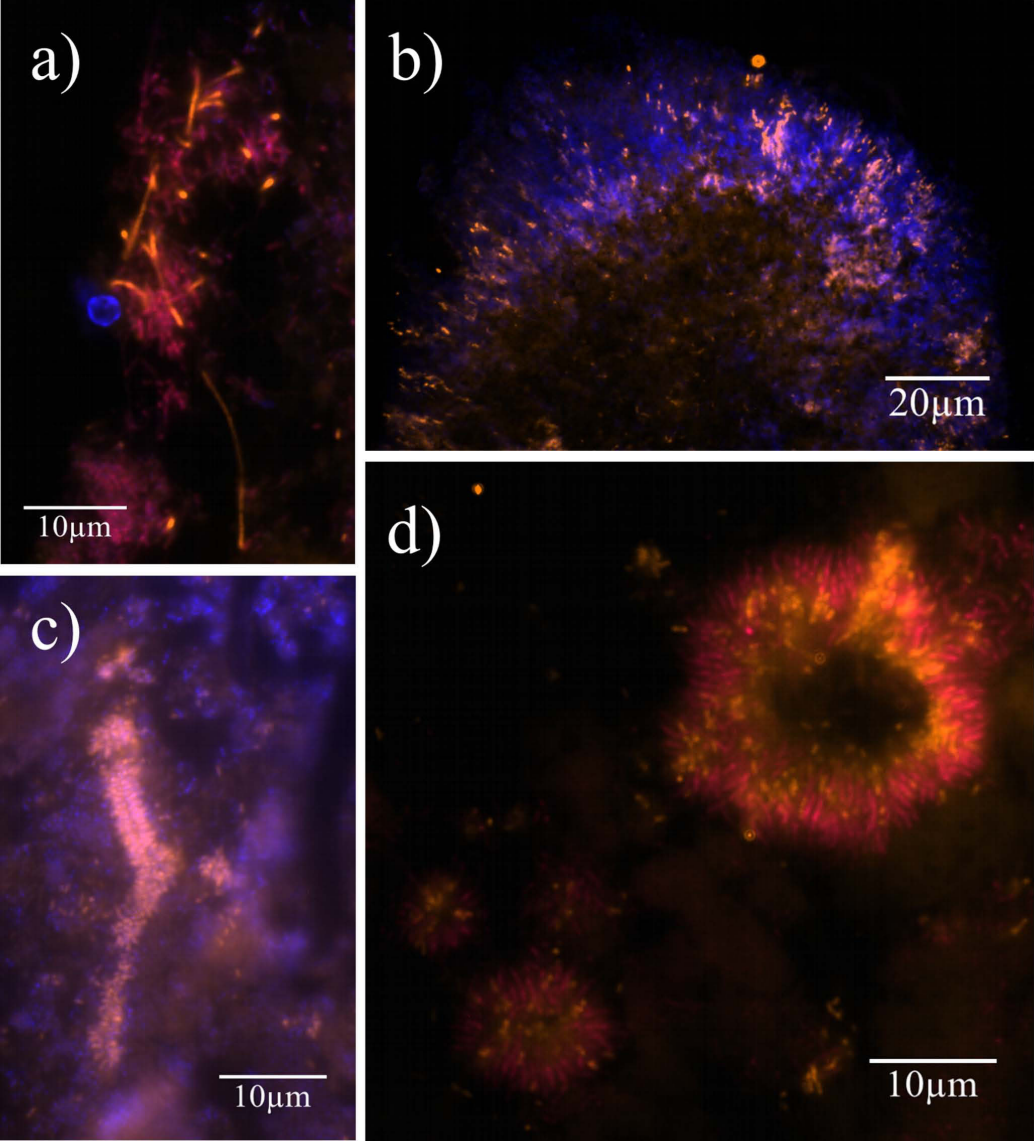
**Formations of *F. alocis *in carrier-borne biofilms**. FISH on different carriers with GAP biofilms using the probes EUB 338-Cy5 (magenta) and FIAL-Cy3 (bright orange) along with DAPI staining (blue). EUB 338 detects the whole bacterial population while FIAL visualizes *F. alocis *specifically. DAPI stains both bacteria and host cell nuclei. High magnifications show *F. alocis *in different areas of the biofilms. (a): Overlay of Cy3, Cy5 and DAPI filter sets. In some regions of the biofilm *Filifactor *rods can reach a considerable length. (b and c): Overlay of Cy3 and DAPI filter sets. (b) shows the radial orientation of *F. alocis *and other organisms on the surface of a mushroom-like protuberance of the biofilm. (c) shows *F. alocis *forming test-tube-brush-like structures around a signal-free channel. (d): Overlay of Cy3 and Cy5 filter sets. *F. alocis *and fusiform bacteria form concentrical structures.

In the carrier-grown biofilms, the organism could be visualized in those areas that had grown in the depth of the pocket, but rarely in areas corresponding to the cervical part of the pocket and rarely on the very tip of the carrier. In most cases, *Filifactor *colonized the side of the carrier facing the soft tissue (Figure 4c) and could only be found in few numbers or not at all on the carrier side facing the root (Figure 4b). Many parts of the biofilm showed *F. alocis *as a short rod of 1-2 μm length, whereas at some sites the organism appeared longer, extending to 7-8 μm (Figure 5a). While in some areas *Filifactor *cells seemed to be scattered within the biofilm without any recognizable pattern, numerous sites clearly showed a higher degree of organisation. Repeatedly, *F. alocis *could be found in densely packed groups (Figure 4c), arranged in concentrical structures (Figure 5d) or grouped in "test-tube brush" formations [43] around signal free channels (Figure 5c). Figure 5b shows the radial orientation of *F. alocis *towards the surface of a mushroom-like protuberance of the biofilm.

**Figure 6:**
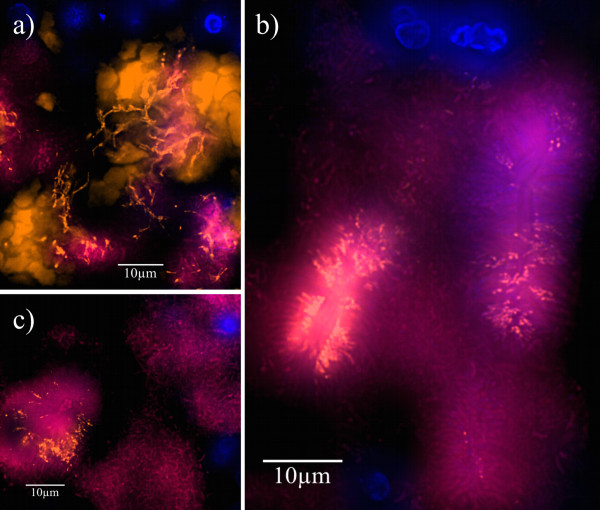
**Formations of *F. alocis *in periodontal tissue**. FISH on a biopsy gained during periodontal surgery using the probes EUB 338-Cy5 (magenta) and FIAL-Cy3 (bright orange) along with DAPI staining (blue). EUB 338 visualizes the entire bacterial community, while FIAL detects only *F. alocis*. DAPI stains both host cell nuclei and bacteria. High magnifications depict *F. alocis *in different parts of the biopsy. (a): *F. alocis *forms tree-like structures among coccoid and fusiform bacteria and autofluorescent erythrocytes. (b) shows *F. alocis *forming palisades with fusiform bacteria around large rodshaped eubacterial organisms. (c) shows *F. alocis *being part of concentrical bacterial aggregations resembling those detected in GAP carriers.

Similar formations that indicate ultrastructural organisation of the biofilm could be observed in the gingival biopsy. In several areas, *F. alocis *formed branch-like structures within the affected tissue (Figure 6a) or palisades around large rodshaped bacteria (Figure 6b). Again, *Filifactor *was observed among the organisms in concentric bacterial aggregations (Figure 6c).

## Discussion

To our knowledge, the present study is the first to analyse the prevalence of *F. alocis *in samples from both GAP and CP patients, and subjects with apparent periodontitis resistance. The detection of the organism in 77.8% of the GAP patients and in 76.7% of those suffering from CP is convincing evidence that suggests an involvement of *F. alocis *in periodontal disease. Equally striking is the low prevalence of *Filifactor *in the PR group. All of these patients had reached the age of 65 years and were in good periodontal condition without the help of extensive therapeutic efforts. Even if a multitude of factors including oral hygiene and immune response contributed to their periodontal status, one would assume that frequent detection of an organism in the GAP and CP groups along with scarce detection in PR patients, as is the case for *F. alocis*, indicates pathogenic rather than commensal behaviour.

One can argue that deep periodontal pockets harbour increased numbers of bacteria and that any organism inevitably should be isolated more constantly from CP patients (mean pocket depth: 7.13 mm, 1.4 mm SD) and especially GAP patients (7.81 mm, 2.48 mm SD) than from PR patients (3.63 mm, 0.79 mm SD). However, dividing the entirety of the sampled sites into four groups according to the measured PPD (I: 1-3 mm, II: 4-6 mm, III: 7-9 mm, IV: > 9 mm), statistical analysis of pockets between 4 and 6 mm still reveals a significantly higher prevalence of *F. alocis *in both the GAP and the CP group compared to the PR group. In addition, the organism was not detected significantly more frequently in deeper pockets (7-9 mm) than in rather shallow pockets (4-6 mm) in both GAP and CP patients. Although a connection between PPD and bacterial load cannot be denied, these findings indicate that the influence of pocket depth does not invalidate the aforementioned results.

If one compares the prevalence rate of *F. alocis *to those of the widely accepted periodontal pathogens *P. gingivalis*, *P. intermedia*, *A. actinomycetemcomitans*, *T. denticola*, *F. nucleatum*, and *T. forsythia *(see Figure 2b), investigated in these very samples using identical methods, *Filifactor *is the third most prevalent for GAP and second most prevalent for CP patients and is thus at eye level with organisms that are considered key players in periodontal disease. At the same time, *F. alocis *shows the lowest prevalence in the PR group of all analysed organisms. Together with *F. nucleatum*, *F. alocis *is the only organism to show a significantly higher detection frequency in both GAP and CP patients compared to the PR group.

Using PCR-based identification methods may introduce bias, since structurally different organisms could exhibit different copy numbers of ribosomal genes and will generally respond differently to DNA isolation and the chosen set of broad range bacterial primers [44]. However, the relevance of *F. alocis *is supported by several other epidemiological studies conducted in the past years using DNA-based techniques. *F. alocis *was detected in GAP patients as well as in CP patients with prevalence rates varying between 45% [29] and 90% [28], depending on the methods employed. Some authors propose *F. alocis *as a marker organism for periodontal disease [28] and even for the shift from periodontal health to disease [19].

Our data strongly support the findings of these studies and motivated the attempt to visualize *F. alocis *within the periodontal biofilm of GAP patients using FISH. The organism could be detected in high numbers in the majority of the examined carriers. The percentage of positive patients approximately matches the dot blot results. Strikingly, several areas of the biofilm show *F. alocis *in densely packed groups (Figure 4c) or as a part of concentric bacterial agglomerations (Figure 5d) - formations that suggest a certain degree of organisation to the observer. Moreover, the organism could be visualized in structures that are considered characteristic architectural features of periodontal biofilms. *F. alocis *is among the bacteria in mushroom-like protuberances on the surface of the biofilm (Figure 5b) and it contributes, grouped around what might be diffusion or convection channels, to the formation of structures reminding of test-tube brushes (Figure 5c). The close colocalization of *F. alocis *with other periodontal pathogens suggests that *Filifactor *might be involved in coaggregation events that take place during the establishment and maturation of the biofilms and that are thought to play a crucial role in biofilm formation [45]. Moreover, the tight colocalization might indicate necessary symbiotic relationships that could help to explain the fastidiousness of *Filifactor*.

Just like group I treponemes [31], *F. alocis *predominantly colonizes the apical and middle third of the carriers and could only casually be detected in the cervical third. Most interestingly, the organism preferably settles on the side of the carrier facing the soft tissues and is thus in immediate contact to the host's immune defence. All these observations point to a causal involvement of *F. alocis *in the formation and maintenance of the analysed biofilms.

However, one might question whether these carrier-borne biofilms accurately model the unperturbed biofilms in periodontitis patients. Wecke et al. [31] compared the bacterial load after 3 and 6 days and showed that the biofilm mass covering the carriers increases with time. The presence of *F. alocis *on only one side of the membranes is further evidence that these samples are not simply fragments of biofilm torn out of the pocket during the removal of the carriers, but in fact newly grown biofilms that form while the carriers are in situ. Although FISH reveals structural elements specific to periodontal biofilms, one cannot deny that the introduction of the carrier into the periodontal pocket creates an artificial environment. The barrier between root surface and pocket epithelium might hamper access of the immune system to the bacteria on the tooth side, while only the biofilm growing on the soft tissue side actually faces the host. Moreover, these biofilms do not form on natural substrate but instead on ePTFE membranes. However, it seems likely that the substrate is of minor importance to the biofilm development. Wecke et al. [31] did not observe differences between biofilms grown on different carrier materials, and it is likely that the acquired pellicle, which covers both the root and the membrane, renders colonization conditions on a broad range of materials alike. This claim is supported by microscopic examination of the biopsy submitted to FISH. *F. alocis *could be visualized in high numbers and detected in arrangements similar to those seen in carrier-borne biofilms. Thus, a contribution of *Filifactor *to the structural organisation of 'naturally' grown biofilms seems highly probable.

The applied carrier system proves to be a valuable tool for the exploration of periodontal biofilms as it allows to investigate topographic relations within the pocket without invasive treatment. Subsequent FISH permits to analyse the distribution and colocalization of potential pathogens within the biofilm and can thus contribute to a better understanding of the complex host-microbe interactions that lead to periodontal destruction.

## Conclusions

The prevalence of *Filifactor alocis *in both GAP and CP patients was found to be elevated as compared to PR control. *F. alocis *thus seems to be a powerful diagnostic marker organism for periodontal disease. FISH revealed the involvement of *F. alocis *in numerous structural arrangements that point to its potential role as one of the architects of structural organisation within periodontal biofilms. *Filifactor alocis *should be considered an important periodontal pathogen and warrants further research.

## Authors' contributions

SS assisted in designing the study, designed and optimized the oligonucleotide probe FIAL, participated in patient sample preparation, carried out dot blot and fluorescence in situ hybridizations, evaluated the data and drafted the manuscript. BR collected patient samples for dot blot hybridization, performed statistical analysis and helped to draft the manuscript. ALG provided the initial idea and participated in designing the study. AP participated in patient sample preparation, dot blot hybridizations and FISH probe optimization. JH provided the gingival biopsy, participated in patient sample preparation and FISH experiments. MB assisted in probe design and dot blot hybridizations. AF developed the periodontal carriers and collected subgingival biofilms for FISH experiments. UBG was involved in designing the study and supervised the work. AM designed and supervised the study and the experiments, analysed the data and participated in writing. All authors read and approved the final manuscript.

## Supplementary Material

Additional file 1 **Optimization of probe FIAL for FISH using the program daime**. FISH was performed incubating fixed cells of *F. alocis *and *F. villosus *with different hybridization mixes. Signal intensities (Relative fluorescent Units, RU) emitted by *F. alocis *and *F. villosus *at different formamide concentrations were calculated from images taken with a fixed exposure time. Due to unspecific binding of FIAL, the light emission of *F. villosus *cells remained below 50 RU at every level of formamide. The signal emitted by *F. alocis *cells was considered sufficient using formamide concentrations of up to 20% (v/v). Click here for file
