# Supplementary figures and images for: Filifactor alocis - involvement in periodontal biofilms
Source: BMC Microbiol. 2010 Mar 1;10:66. doi: 10.1186/1471-2180-10-66 (PMC2846919; doi:10.1186/1471-2180-10-66)

## Slide 1
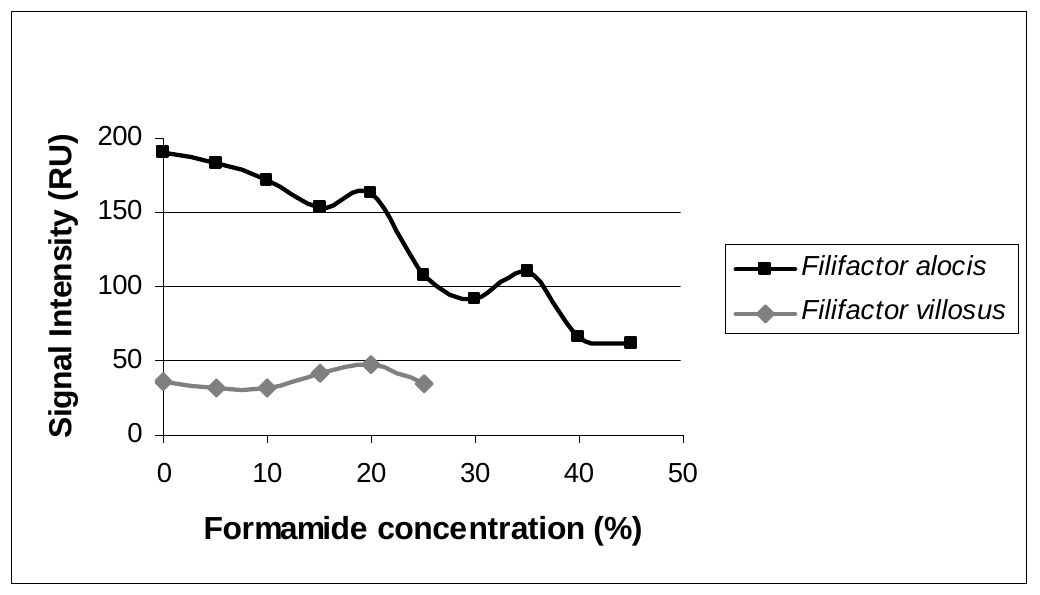

Supplement: Additional file 1 — Optimization of probe FIAL for FISH using the program daime. FISH was performed incubating fixed cells of F. alocis and F. villosus with different hybridization mixes. Signal intensities (Relative fluorescent Units, RU) emitted by F. alocis and F. villosus at different formamide concentrations were calculated from images taken with a fixed exposure time. Due to unspecific binding of FIAL, the light emission of F. villosus cells remained below 50 RU at every level of formamide. The signal emitted by F. alocis cells was considered sufficient using formamide concentrations of up to 20% (v/v). [file 1471-2180-10-66-S1.PPT]
